# Virtual Seminar on Coronavirus 2019 for the US-Mexico Border Region: Building Opportunities for Communication and Collaboration

**DOI:** 10.1007/s10903-021-01190-y

**Published:** 2021-04-21

**Authors:** Benjamin Aceves, Nicolas Lopez-Galvez, Gudelia Rangel, Eduardo Gonzalez-Fagoaga, Rogelio Zapata-Garibay, Cecilia Rosales

**Affiliations:** 1 Division of Public Health Practice and Translational Research, Mel and Enid Zuckerman College of Public Health, University of Arizona, 550 E. Van Buren Street, UA Phoenix Plaza Building 1, Phoenix, AZ 85724 USA; 2 Social Interventions Research and Evaluation Network, University of California, San Francisco, CA USA; 3 Department of Environmental Health Sciences, Mel and Enid Zuckerman College of Public Health, University of Arizona, Tucson, AZ USA; 4 US-Mexico Border Health Commission, Tijuana, B.C Mexico; 5 El Colegio de La Frontera Norte, Tijuana, B.C Mexico

**Keywords:** Border region, COVID-19, Collaboration, Epidemiology, Migrant

## Abstract

While the US-Mexico border region has had increasing restrictions due to coronavirus 2019 (COVID-19), the economically and socially integrated region continues to facilitate necessary movement between the two countries. Binational partners representing universities, government, and health delivery worked together to develop a COVID-19 Virtual Seminar for the US-Mexico Border Region, which consisted of weekly sessions in Spanish designed to better facilitate communication and collaborative systems between border states. In total 835 participants registered for the virtual seminar with attendance ranging from 394 in Session 1 to 269 in Session 6. From evaluation surveys (n = 297), organizers observed a large plurality of healthcare professionals, followed by students, researchers, and government employees. The seminar’s contribution to increasing collaborative and communication systems identified major needs in the region surrounding surveillance and monitoring; increased resources for migrant shelters to control outbreaks; an increase in personal protective equipment; tracking binational cases.

## Background

Coronavirus-19 (COVID-19) has had a major impact on population health and health systems across the globe, with the WHO reporting approximately 108.2 million cases and over 2.3 million deaths globally [1]. Government entities across the global have recently been confronted with the critical preparedness associated with COVID-19. The rapid spread of the virus called to action decision-makers at every level to monitor and limit the transmission of the virus. Responses to the COVID-19 have varied across countries and regions, ranging from school and university closures, restrictions on travel, prohibition of public gatherings, extension of social welfare programs, active transmission monitoring, among other public health interventions [2]. These actions have been in line with several guidelines recommended by the World Health Organization in identifying effective approaches and implementing actions that enhance transmission prevention [3]. However, these guidelines may at times differ from other existing guidelines or actions taken by federal governments. This has caused a particularly difficult and unique situation for states governments located in the US-Mexico border region, given the presence of two federal governments.

The US-Mexico border region stretches for approximately 2000 miles along the southern end of the US and most northern area of Mexico. It consists of the US states: California, Arizona, New Mexico, and Texas; and the Mexican states: Baja California, Sonora, Chihuahua, Coahuila, Nuevo Leon, and Tamaulipas. The interdependence of the two countries has continued manifest into a tightly woven region that shares economic, cultural, social, and behavioral ties, regardless of the increased politicization of the border region in recent decades [4]. Overall, dynamic movement between the two countries has helped shape its current economic success and the cultural identity of the region. In this addition, this movement has resulted in a shared risk for the spread and transmission of the COVID-19. While, attempts have been made by both the US and Mexico to limit non-essential travel, continued connectivity is essential for the economic and well-being of the region [5]. This has resulted in a need for collaborative action by state health departments and ministries who must actively work to decrease transmission, and coordinate with their counterpart.

In order to better facilitate communication and collective action to monitor and prevent COVID-19 across border region, the US-Mexico Border Health Commission, the University of Arizona, and El Colegio de la Frontera Norte established a virtual seminar series. The series conducted in Spanish shared the state of COVID-19 along the border region, public health prevention efforts, and actions taken by every state government to mitigate the impact of the virus. The seminar series was specifically designed for health personnel and providers, academics, students, among others interested in the topic. The overall objectives of the seminar series to promote information exchange, enhance monitoring processes, identify mutual issues,and promote discussions surrounding solution-based approaches.

## Seminar Format and Logistics

The COVID-19 Virtual Seminar Series for the US-Mexico Border Region was designed as six weekly 90-min sessions with experts in the field invited to present for approximately 20 min.[6] The format of the session consisted of an introduction by the organizers, facilitation of presentations by border health experts, and response to questions posed by attendees. The topics of each session and corresponding dates are listed in Table 1.

**Table 1:** Session Topics and Dates

Session	Dates	Topic
1	April 30, 2020	Epidemiology of COVID-19 in US and Mexico
2	May 7, 2020	Addressing the epidemic in the Tamaulipas-Nuevo León-Coahuila-Texas
3	May 14, 2020	Addressing the epidemic in Chihuahua-New Mexico
4	May 21, 2020	Addressing the epidemic in Sonora-Arizona border region
5	May 28, 2020	Addressing the epidemic in the Baja California-California border region
6	June 4, 2020	Discussion with state decision-makers

### Participants and Data Collection

All sessions were conducted in Spanish using Zoom with only presenters having permission to share video and audio, while participants had access to a chat feature for questions, reflections, and/or comments on the seminar. Participants were asked to register prior to sessions. All registered participants were able to attend all sessions, given they were subsequently were notified and provided information for every weekly session. At the conclusion of the seminar series, registered participants received an online evaluation form. As no personal or identifiable information was collected, and the investigators are reporting the findings of an open public seminar, US-Mexico Border Health Commission exempted from review.

## Series Summary

As seen in Table 1, session 1–5 began by providing an epidemiological picture of COVID-19 in the border region and then later each border state. Across sessions states emphasized their efforts in collaborating with health systems across their region, along with other public and private entities. In addition, states consistently stressed a need for additional personal protective equipment and additional resources in hospitals to care for patients. Several Mexican states also called for training in hospital care, as well as the increased of monitoring and medical attention to the migrant population located in migratory stations, repatriation and shelters, in collaboration with the Mexican—National Institute of Migration. These was also seen to have major impact in border states with significant agricultural communities such as Arizona, Texas, and California. Lastly a major finding was the identification in binational cases–individuals who were transmitted the virus on one side of the border and sought health services on the other side.

The last seminar brought together decision-makers across the region to discuss issues across states in the US-Mexico border region, along with collaborative efforts to increase surveillance and control of COVID-19. Identified challenges discussed across the US-Mexico border region:

- Surveillance and monitoring of suspicious and positive cases, along with tracking of contacts to decrease the spread of the virus
- Increasing the diagnostic capacity
- Acquiring of supplies including personal protective equipment for essential workers and healthcare workers.
- Strengthening and standardizing of mental health care programs
- Controlling the outbreaks among vulnerable populations specifically, indigenous communities and migrant farm workers
- Identification and tracking of cases, as well as exchange of information at the binational level, particularly to address the transmission through binational cases.

### Participants

As seen in Table 2, 835 people registered in the seminar of which 297 (35.6%) answered the seminar evaluation questionnaire. Table 3 demonstrates there was a greater participation (86.5%) of people from the Mexican side of the border, mainly from the six neighboring states. In contrast, there was a low participation of people residing in the border states of the United States (11.8%), which could be due to the fact that the seminar was only held in one language, Spanish. Another aspect to highlight is the participation of people from third countries, such as Denmark, Brazil, El Salvador and Peru. As seen in Appendix 1 under the additional results from the evaluation questionnaire (n = 297), overall participants were very satisfied with the seminar series with approximately 94% rating the session as very good or excellent. Many participants noted that it was quality of information and the opportunity to learn that factored into their rating of the sessions.

**Table 2:** Session Summary

Total seminar participants registered	835	
Attendees Per Session		
Session 1	394	47.2%
Session 2	353	42.3%
Session 3	326	39.0%
Session 4	308	36.9%
Session 5	286	34.3%
Session 6	269	32.2%

**Table 3:** Participant Evaluation

Total number of evaluation respondents	297
Percentage of registered respondents	35.6%
Country	
Mexico	86.5%
US	11.8%
Other	1.7%
State	
Mexican border state	77.4%
US border state	12.7%
Mexican non-border state	9.6%
US non-border state	0.3%

## Discussion

Given the fluidity of the border region, both in crossing of people, goods and services, the Virtual Seminar highlighted the importance of continued communication and collaboration in impeding the epidemic in the US-Mexico border region. As most countries around the world, the US and Mexico, were not an exception to the unpreparedness in addressing the magnitude of this pandemic.

In 2011, an infusion of resources was distributed to states across the US and Mexico through the US-Mexico Border Health Commission, which was establish and approved by both federal governments for prevention efforts and public health emergencies [7, 8]. These resources afforded the US and Mexico border states enhancement of their existing and longstanding collaborative efforts at the state and local levels [9]. In 2003, the Early Warning Emergency Response Disease Surveillance (EWIDS) program was developed by CDC, “to provide rapid and effective laboratory confirmation, and to expand surveillance capabilities.”[10] However with the increasing political polarization of the border region and immigration, over time these critical programs have lost critical funding. The has severely impacted current efforts to control COVID-19. The funding must be increased to further combat the virus in the region and ensure that the health of the nation is prioritized to prevent further COVID-19 outbreaks in these border states.

### New Contributions and Recommendations

In addition, there still lacks a cohesive effort by border states to collectively define binational cases. As such, there is an opportunity to recommend at the very least the importance of revisiting collective binational surveillance efforts at both federal and state levels, regardless of political affiliation. These cases that move seamlessly back and forth across the international boundary need to be properly and promptly addressed to curve current pandemics along the border region. This also has critical implications for contact tracing—improved implementation of communication and surveillance systems can immediately identify binational cases through contact tracing to rapidly mitigate community spread on both sides of the border. In addition, this can be extended to migrant shelters and communities that have seen consistent outbreaks, and are in urgent need of additional resources to combat COVID-19 transmission. Lastly, the Operational Protocol for Binational Communication and Coordination between Mexico and the United States on Disease and Outbreak Notifications should be updated, implemented, and serve as policy for improving practice in communication sharing and forging opportunities for collaboration with the current pandemic and future events that require early detection and response.

## Appendix 1

See Table 4

**Table 4:** Additional Results from the Evaluation Questionnaire (n = 297)

How did you find out about the virtual seminar?	
I received a digital invitation addressed to me	13.1%
I received a digital invitation addressed to the general public	30.0%
A colleague or co-worker shared the information	37.7%
Other	19.2%
How was the registration process?	
Very simple	68.4%
Simple	29.0%
Not simple	2.4%
Complicated	0.3%
How the process to connect to each session?	
Very simple	66.7%
Simple	30.6%
Not simple	2.0%
Complicated	0.7%
In general, how would you rate the session(s) you attended?	
Excellent	60.9%
Very good	33.3%
Good	5.4%
Normal	0.3%
What was the main reason for the rating you assigned for session(s) attended?	
Quality of information presented and opportunity to learn more	79.1%
Quality of presentors	16.2%
Time dedicated to the topics presented	3.7%
Session duration	0.7%
Time dedicated to address questions or concerns	0.3%
